# Body fat percentage, cardiorespiratory fitness and arterial blood pressure in children and adolescents: a longitudinal analysis

**DOI:** 10.1186/s12872-022-02704-8

**Published:** 2022-06-15

**Authors:** Caroline Brand, Ana Paula Sehn, Camila Felin Fochesatto, João Francisco de Castro Silveira, Jorge Mota, David Martinez Gomez, Anelise Reis Gaya, Cézane Priscila Reuter, Jane Dagmar Pollo Renner

**Affiliations:** 1 Graduate Program in Health Promotion, University of Santa Cruz do Sul (UNISC), Independência Av, 2293 – Bloco 42, s. 4206, RS Santa Cruz do Sul, Rio Grande do Sul, Brazil; 2 School of Physical Education, Physiotherapy and Dance, Federal University of Rio Grande do Sul, Felizardo Street, 750, Porto Alegre, Brazil; 3 Faculty of Sport, University of Porto, Dr. Plácido da Costa St, Porto, Portugal; 4 Department of Preventive Medicine and Public Health, Universidad Autónoma de Madrid/IdiPaz, CIBER of Epidemiology and Public Health (CIBERESP), Madrid, Spain; 5 IMDEA Research Institute on Food & Health Sciences, Campus of International Excellence (CEI), Universidad Autónoma de Madrid (UAM) + Spanish National Research Council (CSIC), Madrid, Spain

**Keywords:** Adiposity, Fitness, Systolic blood pressure, Diastolic blood pressure, Youth

## Abstract

**Background:**

A better understanding of how cardiorespiratory fitness (CRF) and adiposity interact to associate with arterial blood pressure over time remains inconclusive. Thus, the aim of the present study was to examine whether changes in CRF moderates the association between body fat percentage (BF%) and arterial blood pressure in children and adolescents.

**Methods:**

This is an observational longitudinal study with 407 children and adolescents aged 8–17 years followed-up for three years from a city in Southern Brazil. Participants were evaluated in 2011 and 2014. CRF was measured by validated field-based tests following the *Projeto Esporte Brazil* protocols and peak oxygen uptake (VO_2peak_) was estimated. BF% was determined by the measures of tricipital and subscapular skinfolds using equations according to sex. Systolic and diastolic blood pressure (SBP, DBP) were measured with a sphygmomanometer according to standard procedures. Moderation analyses included multiple linear regression models adjusted for sex, age, pubertal status, height, socioeconomic level, skin color, and the arterial blood pressure variable itself at baseline.

**Results:**

It was observed a significant inverse association between VO_2peak_ at baseline with SBP (β = − 0.646 CI95% = − 0.976  − 0.316) and DBP (β = − 0.649 CI95% = − 0.923  − 0.375) at follow-up and a positive association between BF% at baseline with SBP (β = 0.274; CI95% = 0.094 0.455) and DBP (β = 0.301; CI95% = 0.150 0.453) at follow-up. In addition, results indicated a significant interaction term between changes in VO_2peak_ and BF% at baseline with both SBP (p = 0.034) and DBP at follow-up (p = 0.011), indicating that an increase of at least 0.35 mL/kg/min and 1.78 mL/kg/min in VO_2peak_ attenuated the positive relationship between BF% with SBP and DBP.

**Conclusion:**

CRF moderates the relationship between BF% and SBP and DBP in children and adolescents.

## Introduction

The prevalence of hypertension has increased worldwide in the last years, affecting not only the adult population anymore. Evidence of a systematic review and meta-analysis show a global estimation prevalence of 4% and 10% of children and adolescents presenting hypertension and prehypertension, respectively [1]. More specifically, approximately one in ten Brazilian adolescents are estimated to present hypertension [2] and previous findings have also demonstrated higher levels of systolic and diastolic blood pressure (SBP and DBP) in Southern Brazilian children and adolescents compared to international reference values [3]. This alarming growth trend has become a more common public health already during childhood and adolescence and therefore, there is a need to start efforts on preventing hypertension development as early as possible by the identification of associated factors.

Despite multifactorial origins [4], hypertension non-pharmacological management for pediatric populations includes weight loss and physical exercise in addition to dietary intervention and stress control [5]. Indeed, the literature has suggested that hypertension prevalence can be linked to obesity [2] and that physical activity engagement confers benefits for arterial blood pressure [6]. Additionally, there is evidence that a more favorable cardiorespiratory fitness (CRF) level, a marker strictly connected to physical activity practice, is inversely associated with blood pressure [7]. Cross-sectional evidence highlighted that body fat was positively and CRF inversely associated with higher arterial blood pressure levels [8]. However, a better understanding of how CRF and adiposity interact to associate with arterial blood pressure over time remains inconclusive. Thus, an analysis of these interactions through a longitudinal approach could help prioritize targets to prevent, manage, and treat hypertension development at an early age as well as later in life. Therefore, the present study aims to examine whether changes in CRF moderate the association between body fat percentage (BF%) and arterial blood pressure in children and adolescents.

## Methods

### Study design and sample

**Fig. 1:**
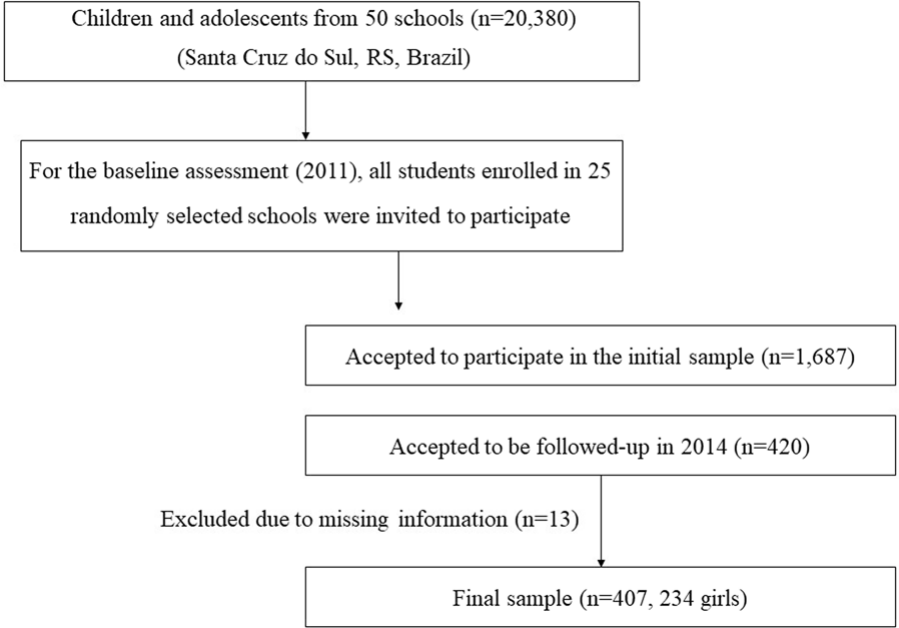
Population and sample design flowchart

This is an observational longitudinal study with participants from the Schoolchildren’s Health Study, which began in 2011. All children and adolescents enrolled in 25 randomly selected public (municipal and state) and private schools from Santa Cruz do Sul, Brazil, were invited to participate in the baseline assessment to be part of a cross-sectional study (1,687 children and adolescents) [9, 10]. All individuals were invited to participate in the follow-up assessment in 2014, only 420 participants accepted to be followed-up (24.9% retention), however, 13 participants were excluded due to missing information, totaling 407 participants at follow-up, aged from 8 to 17 years (Fig. 1).

This study was approved by the University of Santa Cruz do Sul research ethics committee (nº 1.836.983) and it was conducted following Resolution 466/2012 of the National Council of Health in Brazil. The schoolchildren’s parents or legal guardians signed free and informed consent forms.

### Measures

All measurements were taken at University of Santa Cruz do Sul at baseline (2011) and follow-up (2014) periods. Arterial blood pressure was measured with a sphygmomanometer with appropriate brachial perimeter and a stethoscope were placed on their arm. Then, SBP and DBP were determined by manual auscultation, with the student sitting, resting for five minutes prior to measurement, which was made early in the morning. Each device had three different sized cuffs so that researchers could select the most suitable for each arm circumference. Two measurements on the right arm were made, and the lowest blood pressure recorded. All procedures were adopted following the VI Brazilian Guidelines for Hypertension [11].

The BF% was determined through the measures of tricipital and subscapular skinfolds, evaluated using a Lange® caliper (Beta Technology Inc, Houston, TX) by the same evaluator at both the 2011 and 2014 time points. Each skinfold was evaluated twice, and if the difference between measurements was higher than 2 mm, a third evaluation was performed. The lowest value was used for analyses. The BF% was calculated using equations of Slaughter et al. [12] according to sex.

The CRF was assessed by the 9-min running and walking cardiorespiratory fitness test in 2011, described by *Projeto Esporte Brasil* [13], and by the 6-min running and walking CRF test in 2014, described by *Projeto Esporte Brasil* [14]. The indirect submaximal exercise tests, assessed in meters, were used to estimate peak oxygen uptake (VO_2peak_) by the following equations: *9-min test → VO*_*2peak*_ = *47.547* + 0.008 * (Test) – 0.805 * (BMI) + 4.236 * (Sex) [15]; and *6-min test → VO*_*2peak*_ = *41.946* + 0.022 * (Test) – 0.875 * (BMI) + 2.107 * (Sex) [16]; where *test* is the value of meters performed by the student; and *sex* equals to 1 and 0 for boys and girls, respectively.

### Covariates

Information about age, sex, and skin color were obtained through a self-reported questionnaire. Height was measured on the anthropometric scale with a coupled stadiometer. The pubertal status was evaluated at follow-up using Tanner´s criteria [17]. The participant should filled the image corresponding to their current pubertal status considering genital and pubic hair. Therefore, five stages of sexual maturation were considered and classified into pre-pubertal (stage 1), initial development (stage 2), continuous maturation (stages 3 and 4), and matured (stage 5). Socioeconomic status was assessed by the questionnaire of the Brazilian Association of Research Companies [18], considering the head of household’s educational level and the quantity of appliances the family has (car, washing machine, bathroom, among others). A score was obtained according to the answers; thus, the sum of these scores indicated the family’s social class: low (D-E), medium (C), and high (A-B).

### Statistical analysis

Descriptive data are presented as means and standard deviations for continuous variables and absolute and relative frequencies for categorical variables. Initially, we adopted an exploratory analysis according to box-plot visual inspection, scatter-dot graphs to verify the behavior of variables concerning linearity and agreement; also all variables were checked for normality through the Shapiro–Wilk test. The independent student *t*-test was used to verify differences between sexes, whereas the t-test for paired samples was used to verify differences between baseline and follow-up scores. Effect size (Cohen’s d) was calculated. Values of *d* < 0.39 indicated a small difference; 0.40 < *d* < 0.79 indicated a medium difference; and *d* > 0.80 indicated a large difference [19]. Effect sizes (Phi [φ] and Cramer’s V) were also calculated for the chi-squared test, which verified the difference of frequencies between sexes for the categorical variables. Linear regression models were used to test the relationship between baseline values and changes in CRF (VO_2peak_) and adiposity with blood pressure at follow-up. Moderation analyses were tested using multiple linear regression models through PROCESS macro, which is a program extension for the Statistical Package for Social Sciences (SPSS) version 24.0 (IBM Corp, Armonk, NY, USA). The following models were tested: a) associations between changes in CRF (VO_2peak_) with SBP and DBP at follow-up; b) associations between BF% at baseline with SBP and DBP at follow-up; c) Interactions between changes in CRF (VO_2peak_) and BF% at baseline with SBP and DBP at follow-up.

The Johnson-Neyman technique was used to probe interactions by assessing whether changes in CRF (VO_2peak_) moderated the relationship between BF% at baseline with SBP and DBP at follow-up. This technique verifies the association between the independent and dependent variable across different levels of the moderator variable (we present the relationship at 16th, 50th, and 84th percentiles because of the skewness of the moderator variable). In the context of the current study, the technique highlights specific changes in CRF (VO_2peak_) cut point in which the significant relationship between BF% at baseline with SBP and DBP at follow-up appears or disappears. All analyses were adjusted for sex, age, pubertal status, height, socioeconomic level, skin color/ethnicity, and the dependent variable itself at baseline. The probability value p < 0.05 was considered as significant for all analysis.

Multiple linear regression was used as a statistical test for sample calculation on G*Power 3.1 program (Heinrich- Heine-Universität), considering the following parameters: test power (1-β) = 0.95, a significance level of α = 0.05, and effect size of 0.05. The number of predictors considered was 10, and the minimum number of participants was established as 348. However, to avoid probably difficulties with sample loss, an increase of 15% was assumed, totaling 400 children and adolescents.

## Results

The general sample characteristics of the whole sample and stratified by sex are presented in Table 1. Compared with baseline, boys presented higher VO_2peak_ (*d* = 0.20;), SBP and DBP (0.42 < *d* < 0.72;), as well as lower BF% (*d* = 0.40;) at follow-up, while girls showed higher VO_2peak_ (*d* = 0.25;), SBP (*d* = 0.88;) and DBP (*d* = 0.63;) at follow-up. In addition, boys had lower BF% (Baseline: *d* = 0.21; Follow-up: *d* = 0.55; medium difference) and higher VO_2peak_ at both baseline and follow-up (*d* > 0.93; both large differences) than girls. Lastly, boys exhibited a higher decrease of BF% and a higher increase of VO_2peak_ compared to girls, whereas girls exhibited a higher increase of SBP (*d* < 0.39).

**Table 1:** Descriptive sample characteristics at baseline and follow-up

	Boys	Girls	Total	Effect size Cohen’s *d*
	n = 173	n = 234	n = 407	
		Mean (SD)		
Age (Baseline; years)	10.08 (2.10)*	10.01 (2.04)*	10.02 (2.06)	0.03
Age (Follow-up; years)	12.69 (1.99)	12.64 (1.98)	12.66 (1.98)	0.03
Weight (Baseline; kg)	40.63 (14.28)*	39.03 (12.15)*	39.71 (13.10)	0.12
Weight (Follow-up; kg)	54.62 (17.68) †	50.94 (12.64)	52.50 (15.08)	0.25
Height (Baseline; meters)	1.43 (1.36)*	1.42 (1.29)*	1.43 (1.32)	0.01
Height (Follow-up; meters)	1.58 (0.13) †	1.55 (0.10)	1.56 (0.11)	0.26
BF% (Baseline; %)	21.85 (8.02) †*	23.35 (6.10)	22.71 (7.01)	0.21
BF% (Follow-up; %)	19.15 (7.70) †	22.90 (6.00)	21.31 (7.01)	0.55
Δ BF% (T_1_—T_0;_ %)	− 2.69 (6.75) †	− 0.45 (4.98)	− 1.40 (5.90)	0.39
VO_2peak_ (Baseline; mL/kg/min)	47.09 (4.16) †*	41.69 (3.53)*	43.99 (4.65)	1.42
VO_2peak_ (Follow-up; mL/kg/min)	48.07 (6.67) †	42.57 (5.25)	44.91 (6.48)	0.93
Δ VO_2peak_ (T_1_—T_0;_ mL/kg/min)	0.94 (4.58) †	0.86 (3.48)	0.89 (3.97)	0.02
SBP (Baseline, mmHg)	100.03 (11.07)*	98.10 (10.78)*	98.92 (10.94)	0.18
SBP (Follow-up, mmHg)	110.06 (14.84)	109.15 (12.85)	109.53 (13.72)	0.07
Δ SBP (T_1_—T_0;_ mmHg)	10.02 (13.88) †	11.04 (12.51)	10.60 (13.11)	0.08
DBP (Baseline, mmHg)	60.91 (11.15)*	59.53 (10.55)*	60.12 (10.82)	0.13
DBP (Follow-up, mmHg)	66.49 (11.90)	66.74 (10.48)	66.63 (11.09)	0.02
Δ DBP (T_1_—T_0_, mmHg)	5.57 (13.30)	7.20 (11.47)	6.51 (12.29)	0.13

		n (%)		Effect size
Skin color				
White	132 (76.3)	185 (79.1)	317 (77.9)	φ = 0.03
Non-white	41 (23.7)	49 (20.9)	90 (28.1)	
Pubertal status (Follow-up)				
Pre-pubertal	20 (11.6)	20 (8.5)	40 (9.8)	V = 0.09
Initial development	40 (23.1)	53 (22.6)	93 (22.9)	
Continuous maturation (stages 3 and 4)	98 (56.7)	143 (61.1)	231 (59.2)	
Maturated	15 (8.7)	18 (7.7)	33 (8.1)	
Socioeconomic level				
High	72 (41.6)	99 (42.5)	171 (42.1)	V = 0.07
Medium	94 (54.3)	117 (50.2)	211 (52.0)	
Low	7 (4.0)	17 (7.3)	24 (5.9)	

Data are expressed as mean and standard deviation (SD) for continuous variables or as absolute and relative frequency for categorical variables; † denotes difference between sexes calculated using the independent student *t*-test (*p* < 0.05); * denotes statistical differences between follow-up and baseline scores using the *t*-test for paired samples (*p* < 0.05); Effect size for the difference between baseline and follow-up scores: BF%: (Boys: 0.40; Girls: 0.09); VO_2peak_ (Boys: 0.20; Girls: 0.25); SBP (Boys: 0.72; Girls: 0.88); DBP (Boys: 0.42; Girls: 0.63); Δ: Changes between follow-up and baseline scores (T_1_ – T_0_); BF%: Body fat percentage; VO_2peak_: Peak oxygen uptake; SBP: systolic blood pressure; DBP: diastolic blood pressure.; φ: Effect size Phi; V: Cramer’s V

Regression analyses showed a significant inverse association between VO_2peak_ at baseline with SBP and DBP at follow-up and a positive association between BF% at baseline with SBP and DBP at follow-up, after adjustments for sex, age, pubertal status, height, socioeconomic level, skin color, and the arterial blood pressure variable itself at baseline. Also, change in VO_2peak_ was negatively associated with SBP and DBP following the same adjustments, whereas changes in BF% showed no relationship with both arterial blood pressure variables (Table 2).

**Table 2:** Association between baseline values and changes in cardiorespiratory fitness and adiposity with blood pressure at follow-up

	β (CI 95%)			
	VO_2peak_ baseline		BF% baseline	
	Model 1	Model 2	Model 1	Model 2
Follow-up SBP	** − 0.639 ( − 0.096 − 0.030)**	** − 0.646 ( − 0.976 − 0.316)**	**0.276 (0.097 0.455)**	**0.274 (0.094 0.455)**
Follow-up DBP	** − 0.655 ( − 0.928 − 0.382)**	** − 0.649 ( − 0.923 − 0.375)**	**0.304 (0.154 0.454)**	**0.301 (0.150 0.453)**

	ΔVO_2peak_		ΔBF%	
	Model 1	Model 2	Model 1	Model 2
Follow-up SBP	− 0.281 ( − 0.578 0.017)	** − 0.329 ( − 0.628 − 0.029)**	0.056 ( − 0.146 0.258)	0.092 ( − 0.114 0.297)
Follow-up DBP	** − 0.284 ( − 0.853 − 0.031)**	** − 0.318 ( − 0.574 − 0.063)**	− 0.016 ( − 0.187 0.155)	− 0.008 ( − 0.167 0.182)

Bold denotes statistically significant

VO_2peak_: Peak oxygen uptake; BF%: body fat percentage; SBP: systolic blood pressure; DBP: diastolic blood pressure; Model 1: adjusted for sex, age, and arterial blood pressure variable itself at baseline; Model 2: adjusted for sex, age, pubertal status, height, socioeconomic level, skin color, and arterial blood pressure variable itself at baseline. Δ Changes in VO_2peak_ and BF% were calculated by subtracting the results at baseline from those at follow-up

**Fig. 2:**
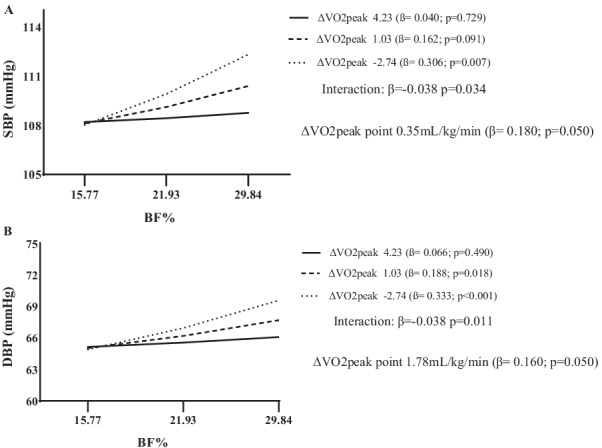
Moderation of cardiorespiratory fitness in the relationship between percentage of body fat and systolic blood pressure (**A**) and diastolic blood pressure (**B**). All analyses were adjusted for sex, age, pubertal status, height, socioeconomic level, skin color and variable in baseline. VO_2peak_: Peak oxygen uptake; BF%: percentage of body fat; SBP: systolic blood pressure; DBP: diastolic blood pressure

Taking into account that changes in VO_2peak_ was negatively associated with both SBP and DBP, and that BF% at baseline presented the same associations, we sought to determine the moderator role of changes in VO_2peak_ in the association between BF% at baseline with arterial blood pressure at follow-up (Table 3). Results indicated a significant interaction term between changes in VO_2peak_ X BF% at baseline with both SBP and DBP at follow-up, indicating that an increase of at least 0.35 mL/kg/min attenuated the positive association between BF% with SBP, while an increase of 1.78 mL/kg/min in VO_2peak_ also attenuated the positive association between BF% with DBP (Fig. 2A and B).

**Table 3:** Moderation of changes in cardiorespiratory fitness in the association between BF% at baseline with arterial blood pressure at follow-up

	β	CI (95%)	*p*
	*SBP follow-up*		
ΔVO_2peak_	0.624	0.170 − 0.269	0.170
BF% baseline	0.201	0.013 0.390	0.037
ΔVO_2peak_ X BF%	** − 0.038**	** − 0.073 − 0.003**	**0.034**
	*DBP follow − up*		
ΔVO_2peak_	0.637	− 0.109 1.384	0.094
BF% baseline	0.228	0.071 0.385	0.005
ΔVO_2peak_ X BF%	** − 0.038**	** − 0.067 − 0.009**	**0.011**

Bold denotes statistically significant

VO_2peak_: Peak oxygen uptake; BF%: body fat percentage; SBP: systolic blood pressure; DBP: diastolic blood pressure; All analyses were adjusted for sex, age, pubertal status, height, socioeconomic level, skin color, and arterial blood pressure variable itself at baseline

## Discussion

Our results indicated that changes in VO_2peak_ moderated the association between baseline BF% and arterial blood pressure at follow-up and might, then, to be protective against the deleterious influence of BF% on the hypertension development in youth. Indeed, an increase of 0.35 mL/kg/min and 1.78 mL/kg/min in VO_2peak_ attenuated the positive relationship between BF% with SBP and DBP, respectively.

According to available evidence CRF and BF% are inversely associated with SBP and DBP, indicating that low CRF levels and high BF% or waist circumference seem to increase the chances of adolescents developing hypertension, present high arterial blood pressure [20–22] as well as higher cardiovascular disease risk [23]. The same results were found when considered high BMI and poor VO_2max_ in the association with arterial blood pressure [24]. In addition, the improvements in CRF presented different results in obesity and normal weight groups, in this sense, it is observed that high CRF influence excessive body weight and cardiovascular disease mortality, being able of promoting improvements in the cardiometabolic risk factors related to obesity, only in obese individuals [25, 26]. Other study demonstrated that CRF seems to be a mediator in the relationship between BF%, BMI, and waist-to-height ratio with cardiometabolic risk factors, indicating that high CRF seems to compensate for the negative effect of adiposity in the cardiometabolic risk factors [27]. In contrast, some studies indicated that high CRF levels seem not to be able to reduce high blood pressure or cardiometabolic risk factors in children [28–30], indicating that is important to maintain normal body weight and high CRF to cardiovascular health [31]. In this context, is relevant to consider the role of pubertal stages, once the maturation process in associated with important hormonal transformation, leading to higher body fat and lower CRF [32].

Therefore, the interactions between BF%, CRF and blood pressure in a longitudinal approach are not well established in the literature, and the present study provide new evidence to fill that gap. Our results provide evidence about the role of increasing CRF levels on attenuating SBP and DBP, even when considering the deleterious influence of adiposity. Excessive adiposity is known to be a strong determinant of high blood pressure in all ages, by leading to metabolic, hemodynamic and inflammatory alterations affecting the heart size and causing left ventricular hypertrophy [33]. In this sense, the higher CRF levels, the lower the chance of hypertension development, even when presenting high BF%. Indeed, our findings are in accordance with the fitness versus fatness paradox, which indicates that CRF may counteract the deleterious influence of adiposity on cardiometabolic health [34]. This findings may be explained by the fact that some mechanisms associated with the benefits of having good CRF levels are capable of reducing the level of chronic systemic low-grade inflammation [35], and also promoting beneficial changes in endothelial function, autonomic nervous system function and insulin sensitivity which in turn may modulate arterial blood pressure relatively early in life [36, 37]. Thus, although the mechanisms explaining the link between CRF and blood pressure are not completely understood, it has been indicated that contributes to maintaining vascular homeostasis, its positively associated with nitric oxide and consequently lower arterial stiffness, as well as the expression of an important regulator of blood pressure, the endothelin-1 gene (a potent vasoconstrictor) is decreased in the presence of high CRF [38–40].

Thus, public health strategies must encourage the increase of CRF levels and not only the reduction of body weight, especially in children and adolescence [41]. Therefore, for the prevention of cardiovascular diseases, multidisciplinary intervention programs should be implemented. In addition, it is important to encourage that children and adolescent achieve at least 60 min per day of moderate and vigorous physical activity in order to enhance CRF levels [42–44].

The main strength of the present study is that we provide an objective recommendation of increasing CRF that children and adolescents should achieve in order to be protected against the deleterious associations of BF% with SBP and DBP (0.35 mL/kg/min and 1.78 mL/kg/min, respectively). A second major strength was the use of a relatively large randomly selected sample of Southern Brazilian school-aged children and adolescents obtained at two time-points across a 3-year time span. In addition, there are few longitudinal studies that demonstrate the association between CRF, BF%, and arterial blood pressure in the pediatric population. However, some limitations also should be pointed out, the use of both 6- and 9-min running and walking tests to evaluate CRF, to minimize this limitation the estimation of VO_2peak_ was calculated. Also, these tests are indirect measure of CRF, although this evaluation is commonly used in many studies [26, 27]. The reliability and validity could have been stronger if gold-standard protocols were utilized, such as VO_2peak_ maximum protocol for CRF and DXA for adiposity assessments. Additionally, the observational design of the present study cannot assert on the causality between CRF, BF%, and arterial blood pressure. Randomized controlled trials are encouraged to properly test if an improvement in CRF or BF% or both will cause improvements on SBP and DBP levels within the pediatric population.

## Conclusion

Changes in CRF moderated the association between baseline BF% with arterial blood pressure at follow-up. Therefore, our findings highlight the need of enhancing CRF levels over time, once it may exert a protective role on arterial blood pressure levels, even when considering the deleterious influence of adiposity.

## Data Availability

The database used and analyzed in the present study is not publicly available as its information may compromise the participants' privacy and consent involved in the research. However, the data are available from the corresponding author, upon request.

## Abbreviations

SBP: Systolic blood pressure
DBP: Diastolic blood pressure
CRF: Cardiorespiratory fitness
BF%: Body fat percentage
VO_2peak_: Peak oxygen uptake
SPSS: Statistical package for social sciences
